# Measures of gait stability: performance on adults and toddlers at the beginning of independent walking

**DOI:** 10.1186/1743-0003-11-131

**Published:** 2014-09-03

**Authors:** Maria Cristina Bisi, Federico Riva, Rita Stagni

**Affiliations:** Department of Electrical, Electronic and Information Engineering “Guglielmo Marconi”, University of Bologna, 2, Viale Risorgimento, 40136 Bologna Italy; Health Sciences and Technologies, Interdepartmental Center for Industrial Research (HST-ICIR), Bologna Italy

**Keywords:** Stability, Gait, Children’s motor development

## Abstract

**Background:**

Quantifying gait stability is a topic of high relevance and a number of possible measures have been proposed. The problem in validating these methods is the necessity to identify a-priori unstable individuals. Since proposed methods do not make any assumption on the characteristics of the subjects, the aim of the present study was to test the performance of gait stability measures on individuals whose gait is a-priori assumed unstable: toddlers at the onset of independent walking.

**Methods:**

Ten toddlers, ten adults and ten elderly subjects were included in the study. Data from toddlers were acquired longitudinally over a 6-month period to test if the methods detected the increase in gait stability with experience, and if they could differentiate between toddlers and young adults. Data from elderly subjects were expected to indicate a stability value in between the other two groups. Accelerations and angular velocities of the trunk and of the leg were measured using two tri-axial inertial sensors. The following methods for quantifying gait stability were applied: stride time variability, Poincaré plots, harmonic ratio, short term Lyapunov exponents, maximum Floquet multipliers, recurrence quantification analysis and multiscale entropy. An unpaired *t*-test (level of significance of 5%) was performed on the toddlers and the young adults for each method and, for toddlers, for each evaluated stage of gait development.

**Results:**

Methods for discerning between the toddler and the adult groups were: stride time variability, Poincaré plots, harmonic ratio, short term Lyapunov exponents (state space composed by the three linear accelerations of the trunk), recurrence quantification analysis and multiscale entropy (when applied on the vertical or on the antero-posterior L5 accelerations).

**Conclusions:**

Results suggested that harmonic ratio and recurrence quantification analysis better discern gait stability in the analyzed subjects, differentiating not only between unstable toddlers and stable healthy adults, but also evidencing the expected trend of the toddlers towards a higher stability with walking experience, and indicating elderly subjects as stable as or less stable than young adults.

**Electronic supplementary material:**

The online version of this article (doi:10.1186/1743-0003-11-131) contains supplementary material, which is available to authorized users.

## Background

Several methods for quantifying the stability of a person during walking (gait stability) have been proposed in the literature [1–5]. These methods have many potential applications in the prevention of falls, especially among elderly subjects and pathologic individuals, e.g. quantifying the risk of fall, allowing quantitative evaluations of prevention and rehabilitation procedures [1]. Synthetic indicators proposed for the quantification of gait stability can be generally grouped into two main classes: stability and variability indices. Stability indices come from mechanical system analysis and, when applied to biomechanics, require some hypothesis about the type of system governing gait control [6]. On the other hand, variability indices aim to evaluate gait stability assuming that high variability represents a manifestation of the system instability [1].

The assumptions made for both stability and variability index definition cannot be easily verified, thus, until now, no method is universally accepted [1, 6]. A possible approach for evaluating these methods is to test their performance on a-priori unstable subjects; in the literature, this performance is usually tested on fallers and non-fallers, referring to fallers as people who experienced falls in the last 6 or 12 months [7]. A limitation of this approach is that data are not collected at the time of the fall, and the fact that a subject is defined as a faller does not necessarily imply that his/her locomotion is always mechanically unstable, in particular during the specific experimental assessment, when both extrinsic (e.g. luminosity, presence of researchers, worn equipment) and intrinsic factors (e.g. health, emotional state, stress) can influence the performance of the subject, modifying his/her natural behavior.

The general purpose of the present study was to test the performance of gait stability measures proposed and applied in the literature [1–5] on individuals who experience more than one fall every day, thus considered unstable by definition: toddlers at the onset of independent walking. Toddlers at their first steps are surely at high risk for falls, and during months of walking experience they fall less and less increasing their stability [8]. With each day of walking, they take more steps, travel farther distances, and fall less: better walkers spontaneously walk more and fall less [9].

It could be argued that toddlers have different characteristics from those of elderly subjects or patients with pathologies. On one hand, it is important to note that methods proposed in the literature do not make any assumption on the characteristics of the subject analyzed, when aiming to quantify the stability of a subject’s motion pattern. On the other hand, given the different characteristics between toddlers and elderly fallers, the goal of the study was not to find reference values for unstable subjects, but to verify if the proposed measures can discern between unstable (toddlers) and stable (young adults) subjects.

In order to technically evaluate the performance of gait stability measures, stable (as reference) and unstable individuals are necessary, thus, a group of young healthy adults and a group of toddlers participated in the study. Moreover, a well performing stability measure, when applied on elderly people, should theoretically give results that indicate a stability level in between unstable subjects (toddlers) and stable ones (young adults) or at least close to the stable group. Thus, in order to evaluate even this aspect of stability measure performance, a group of elderly subjects was included in the study.

The aim of the present work was to estimate the capability of gait stability measures (both stability and variability indices) in differentiating between toddler and young adult groups. Moreover, data from toddlers were acquired longitudinally over a 6-month period in order to test if the analyzed measures were able to follow the increase in gait stability with months of walking experience. Gait stability measures were also applied on a group of elderly subjects to ascertain that they result to be not more stable than young adults.

## Methods

### Study subjects

Ten toddlers (13 ± 2 months, height and weight at 12 months: 77 ± 3 cm, 10 ± 2 kg), ten young adults (27 ± 1 years, 171 ± 9 cm, 67 ± 14 kg) and ten elderly subjects (76 ± 7 years, 168 ± 7 cm, 78 ± 10 kg) participated in the study. All of the toddlers were full-term at birth and had no known developmental delays. All toddlers and adults had no musculoskeletal pathology. Information about each child is shown in Table 1.

**Table 1:** **Analyzed toddler details and trials**

				At birth		At 12 months		
	Age of first steps (months)	Gender	Weeks of pregnancy	Length (cm)	Body mass (kg)	Length (cm)	Body mass (kg)	Trials
1	11	Male	40	54	3,66	77	8,5	T0,T1,T2,T3,T6
2	13	Female	40	50	2,99	75	8,5	T0,T1,T2,T3,T6
3	12	Female	40	52	3,33	80	10,1	T1,T2,T3,T6
4	11	Male	40	52	3,56	81	11,0	T1,T2,T3,T6
5	13	Female	38	47	2,18	76	9,0	T0,T1,T2,T3,T6
6	14	Male	41	55	3,85	79	13,0	T0,T1,T2,T3,T6
7	14	Male	39	50	3,07	79	11,9	T0,T1,T2,T3,T6
8	15	Male	41	50	3,52	77	9,0	T0,T1,T3,T6
9	14	Male	40	48	2,84	73	8,8	T0,T1,T2,T3
10	14	Male	40	50	3,50	75	9,0	T0,T1,T2,T3

The Review Board Committee of the University of Bologna, "Comitato Bioetico", approved this study, and informed consent was obtained from the participants’ parents for toddlers and from adult participants.

Tests on the toddlers were scheduled: during the very first week of independent walking (T0), at month 1 (T1), 2 (T2), 3 (T3), and 6 (T6) after the onset of independent walking.

At each test, parents were asked to answer if, according to their opinion, the child fell less, equal or more than during the period in which the former test was performed. All parents reported a constant decrease of falls from T0 to T6.

Due to illness, holiday and lack of cooperation for 5 toddlers only 4 sessions were available (see detail in Table 1). Since performance of young and elderly adults was not expected to change in a six-month period, one test per adult was scheduled.

### Experimental setup

Two tri-axial wireless inertial sensors (OPALS, Apdm, USA) were mounted using straps respectively on the lower back, at L5 level, and on the right leg, above the lateral malleolus. Sensors characteristics: Accelerometer and gyroscope noise 0.0012 m/s^2^/√Hz and 0.05 deg/s/√Hz respectively, sensors dimensions 48.4 × 36.1 × 13.4 mm (L × W × H), weight <22grams (with battery).

Measures of acceleration and angular velocity of the trunk and of the right leg were recorded (sampling frequency 128 Hz). The participants were asked to walk at self-selected speed in a corridor. When collecting data on toddlers, in order to encourage them to cooperate, moms or nannies called them at the end of the corridor attracting their attention with a toy. Moreover, toddler tests were also video recorded in order to posteriorly check if they either were helping themselves with something (wall, shelves etc.) or were running. In those cases, the identified steps were excluded from the analysis.

### Data analysis

Stride detection was estimated from the angular velocity around the medio-lateral axis of the leg [10]; even if the algorithm proposed by Aminian et al. (2002) was designed for healthy adults with a regular movement pattern, it was adapted and used on toddler data identifying local minima before and after swing phase, which were evident. Stride time was defined as the time elapsed between the first contact of two consecutive footsteps of the same foot.

The first two and last two strides of each test were excluded from the analysis in order to exclude gait initiation and termination phases. For all the participants 10 consecutive strides were analyzed: 14 was the maximum number of strides obtained in the less experienced infants. The potential influence of the available limited number of strides was taken into account depending on the specific index analyzed [11].

The following gait stability measures were calculated for each participant and each test session (see Appendix for a detailed description of each index):

1. Variability indices:
  Stride-time variability [2] (STv). Standard deviation of the stride time.
  Short term (SD1) and long term (SD2) variability of stride time estimated via Poincaré plots [12].
  Harmonic ratio [13, 14] (HR) of L5 acceleration signals. HR was calculated decomposing the whole signal into its harmonics respectively on the vertical (V), antero-posterior (AP) and medio-lateral (ML) axis (HRv, HRap and HRml).
2. Stability indices:
  Short term Lyapunov exponents (sLE) [15, 16]. sLE were calculated using 4 different state spaces compositions: one composed by the three linear acceleration components of the trunk (sLE3) and three composed by the delay embedded state spaces of one acceleration (sLEv, sLEap and sLEml).
  Maximum Floquet multipliers (FM) [6]. FM were calculated using the same 4 state spaces described for sLE: FM3, FMv, FMap and FMml. The mean of all maximum FM at each instant in time was calculated giving an index of the instability over the stride cycle.
  Recurrence quantification analysis (RQA) [17–19]. Calculated indices were recurrence rate (RR), determinism (DET), averaged diagonal line length (AvgL) and maximum diagonal line length (MaxL). All the indices were calculated applying the method on the V, AP and ML accelerations of L5. (Embedding dimension = 5, delay = 10 samples, radius = 40% of the max distance). A radius of 40% was chosen, as suggested by Riley et al. [18], to make sure that RR responded smoothly and was not too high, and that DET did not saturate at the floor of 0 or the ceiling of 100, as approaching these limits would tend to suppress variance in the measure.
  Multiscale entropy (MSE) [20, 21]. MSE was calculated applying the method on the V, AP and ML accelerations of L5 (MSEv, MSEap and MSEml). Consecutively, more coarse-grained time series were calculated on the original data, averaging increasing numbers of data points in non-overlapping windows of length τ. Sample entropy (SE) [21] was then calculated for each coarse grained time series, quantifying the conditional probability that two sequences of m consecutive data points similar (distance of data points inferior to a fixed radius r) to each other will remain similar, when one more consecutive point is included [20]. SE is hence expressed as the negative of the natural logarithm of the conditional probability that two sequences, that are close within a tolerance rδ (where δ is the standard deviation of the original series), form consecutive points remain close at the next point [22]. MSE was calculated for values of τ ranging from 1 to 6, m = 2 and r = 0.2, as suggested by Pincus [23] and later applied by Richman and Moorman to biological time series [21].

For state space reconstruction, on which calculation of sLE, FM and RQA is based, an embedding dimension dE = 5 and a time delay of 10 samples were used, based on previous literature, according to which these parameters are appropriate for the analysis of gait data [7, 15, 24–28].

Raw unfiltered data were analyzed to assure that information was not lost or altered due to filtering. Matlab R2009b (MathWorks BV, USA) was used for data and statistical analysis.

Normal distributions of the estimated parameters for each group were verified using Jarque-Bera test [29]. In the case of toddler data, normality of distributions was verified at each developmental stage analysed. Mean values, and standard deviations of the results for each method were calculated for the young adult group, for the elderly adult group and for the toddler group (at each developmental stage). The mean values obtained for the toddler group at each developmental stage were compared with the mean values obtained from the young adult group during their single session (five comparisons). An unpaired *t*-test with minimum level of significance 5% was performed on the two groups for each method and, for toddlers, for each evaluated stage of gait development. The mean values obtained for the elderly adult group were compared with the mean values of the other two groups in order to evaluate if elderly results were in between very unstable subjects (toddlers) and stable ones (young adults). In order to test if the analyzed measures were able to follow the increase in toddler gait stability with months of experience, an unpaired *t*-test with minimum level of significance 5% was performed between toddler results at T1 and T6.

## Results

Jarque-Bera test results confirmed the normal distribution of the estimated parameters for young adults, elderly adults and toddlers; for toddlers, parameters resulted normally distributed at each developmental stage.

Overall results of the un-paired *t*-test showed that indices allowing to discern between the toddler and the young adult groups were STd, SD1, SD2, HRv, HRap, HRml, sLE3, RRv and RRap, DETv, AvgL_v and AvgL_ap, MaxL_v, MSEv (with τ ≥ 4) and MSEap (with τ ≥ 2). Methods analyzing acceleration data always better discerned the two groups when applied to the V or AP direction. All the mentioned indices, except HRml, AvgL_ap and MSE, when applied to toddler data, showed results that, with months of walking experience, trended towards the adult group, showing higher gait stability, even if no statistical differences between T1 and T6 were found.

When an index allowed differentiating the two groups at the first test (T0 or T1), it was also able to differentiate them at the other developmental stages. HRml, AvgL_ap and MSEap showed almost constant mean values over the six-month period, while MSEv showed results that with months of walking experience diverged from the adult group: the difference between MSEv values at T1 and T6 were not statistically significant.

**Figure 1:**
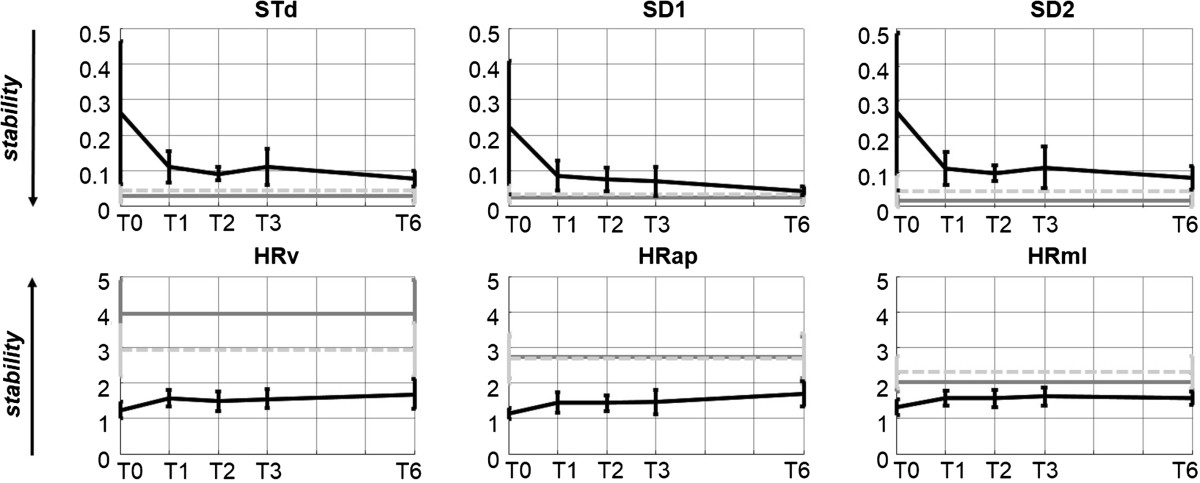
**Variability index results.** Mean and standard deviation of variability indices of toddlers (black solid lines) and adults (dark grey solid lines) and elderly adults (light grey dotted lines). (STv: stride-time variability. SD1 and SD2: short term and long term variability of stride time. HR: Harmonic ratio. V vertical, AP antero-posterior and ML medio-lateral axis).

**Figure 2:**
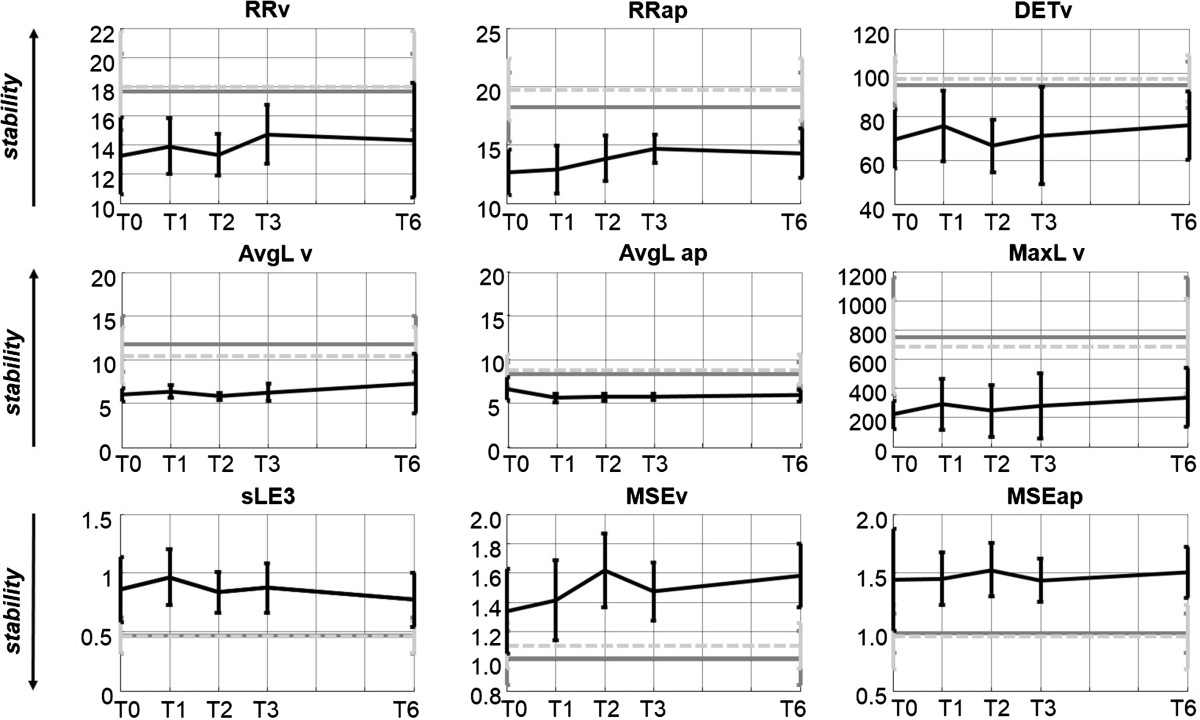
**Stability index results.** Mean and standard deviation of stability indices of toddlers (black solid lines) and adults (dark grey solid lines) and elderly adults (light grey dotted lines). (RR: recurrence rate. DET: determinism. AvgL: averaged diagonal line length. MaxL: maximum diagonal line length. sLE3: Short term Lyapunov exponents calculated using a state space composed by the three linear acceleration components of the trunk. MSE: Multiscale entropy. V vertical, AP antero-posterior and ML medio-lateral axis).

Variability indices calculated on the elderly group always resulted in between young adults and toddlers except for HRml: HRml results indicated elderly as more stable than young adults. Among stability indices, MaxL_v and MSEv indicated elderly subjects as less stable than young adults, sLE3 and MSEap (with τ ≥ 2) as stable as, and RRap, AvgL_ap, more stable than young adults. Mean and standard deviation of the results are shown in Figure 1 (variability indices) and in Figure 2 (stability indices).

sLEv, sLEap and sLEml, FM, RQA and MSE calculated on ML axis did not show statistically significant differences between young adults and toddlers.

## Discussion

In the present work the performance of gait stability measures, proposed in the literature, in differentiating between toddlers at the onset of independent walking and young healthy adults was evaluated. The results of the present study will give an indication on the performance of variability/stability measures: a measure that cannot discern between toddlers at the onset of walking and healthy adults hardly will discern between fallers and non-fallers among elderly people, while measures that can identify toddlers as unstable (or as more unstable than healthy adults) will be more promising. Gait stability measures were also applied on a group of elderly participants as reference: the hypothesis was that stability results should not indicate elderly as more stable than young adults and are expected to show results that are in between very unstable subjects (toddlers) and stable ones (young adults).

All the variability indices applied were able to discern between healthy adults and toddlers, in agreement with what was found generally in the literature [30, 31] and could follow correctly the tendency of toddlers to fall less and less with months of walking experience. HRml was the only variability index that showed higher stability in elderly subjects than in young adults and can thus be excluded from the well performing indices. The choice among the other variability indices could be performed on the basis of index reliability results [11]: when applied on 10 strides, HR indices showed the highest reliability, thus, HRap an HRv could be the best choice.

Among stability indices, sLE3, RQA parameters and MSE calculated on V and AP L5 accelerations showed statistically different results for toddlers and young adults. DETv and RRap indicated elderly subjects as more stable than young adults, and thus could be excluded from the well performing indices. Other indices showed results in agreement with the hypothesis that elderly are as stable as or less stable than young adults. sLE3, AvgL_v and MaxL_v results followed the increasing stability of the toddlers with experience. The choice among the well performing stability indices could be performed on the basis of index reliability results [11]: RQA reaches a steady value when calculated over 10 strides thus, AvgL_v and MaxL_v can be preferred to sLE3.

Even if some indices showed trends converging or diverging from the adult group, no statistical difference between toddler indices at T1 and T6 was found: this could be due to the high variability of toddler indices that, even if resulted to be normally distributed, showed high standard deviations, as it could be expected.

Lyapunov exponents were already applied in the literature to assess stability of toddler gait trajectories, with the aim of analyzing differences between toddlers with typical developments and with Down syndrome [32]: in that study no difference between the two groups was observed. On the other hand, in a previous work by the same group [33] preadolescents with Down syndrome showed larger Lyapunov exponent values than peers with typical development. These results support the findings of this study, where already known stability differences between toddlers and young adults are found by sLE.

FMs were the only methods not able to separate the two groups. Mean FM results for both groups were around 0.55 for FM3, values close to the ones found by van Shooten et al. [31], where also 10 strides were evaluated.

A possible limitation of the present study is the low number of strides analyzed (10), due to both low walking experience and lack of cooperation in toddlers: in literature [11] the minimum number of required strides for each index analyzed in this paper was investigated and results showed that only HR, MSE and RQA reached a steady value when calculated over 10 strides. Thus, a higher number of strides could likely have improved or changed gait stability estimates, in particular for Poincaré Plots and Floquet Multipliers. On the other hand, literature studies, given the difficulties of performing longer data acquisition sessions with elderly and pathologic subjects, often applied them on even lower number of strides (<8) [17, 31, 34].

In this work a simple walking task was chosen for the evaluation, since it is known that majority of fall-related injuries in older adults occur during walking [2]. The experimental setup was minimal (two tri-axial inertial sensors mounted respectively on the lower back and on the right leg): different methods for quantifying stability have been applied to many different biomechanical variables (e.g. joint angles, velocities, temporal parameters, toe clearance etc.) [35], but no standardized setup has been proven to perform better than others. The decision for a minimal setup was guided by the possible applications that an effective simple test with simple setup could have in the prevention of falls, if a good estimator of gait instability is achieved (low cost, portability, velocity of the test etc.). The inertial sensor mounted on the leg was added in order to assure reliable stride detection during the children tests.

## Conclusions

The results of this work suggested that, when using a ten stride walking test, HRv and HRap (among variability indices) and AvgL_v and MaxL_v (among stability indices) result to better estimate gait stability in the analyzed subjects, differentiating toddlers and adults, evidencing qualitatively the expected trends of the toddlers towards a higher stability with months of walking experience and indicating elderly stability in between toddlers’ and young adults’ stability.

## Appendix

### Stride-time variability (STv)

Standard deviation (SD) of stride time was simply calculated as the standard deviation of the stride times in the analyzed time-window [2].

### Short term (SD1) and long term (SD2) variability of stride time estimated via Poincaré plots [12]

Stride time data plots between successive gait cycles, known as Poincaré plots, show variability of stride time data. Statistically, the plot displays the correlation between consecutive stride times data in a graphical manner. Points above the line-of-identity indicate strides that are longer than the preceding, and points below the line of identity indicate shorter strides than the previous ones. The Poincaré plot typically appears as an elongated cloud of points oriented along the line-of-identity. The dispersion of points perpendicular to the line-of-identity reflects the level of short-term variability (SD1) [36]. The dispersion of points along the line-of-identity indicates the level of long-term variability (SD2) [12].

### Harmonic ratio (HR)

The HR was calculated by decomposing acceleration signals into harmonics using a discrete Fourier transform [14]; the summed amplitudes of the first 10 even harmonics were then divided by the summed amplitudes of the first 10 odd harmonics for the AP and V accelerations, and vice-versa for the ML accelerations. This difference is due to the fact that whereas the AP and V accelerations have two periods every stride, showing a dominance of the second harmonic, representing step frequency and subsequent even harmonics, ML accelerations have only one period per stride, reflecting a dominance of the first (and subsequent odd) harmonics [14]. In order to avoid errors that might be introduced by step-detection, HR was not calculated stride by stride, but decomposing the whole signal into its harmonics. A higher HR is an indication of increased smoothness of gait, which can be interpreted as increased stability.

### Short term Lyapunov exponents (sLE)

The first step for local stability analysis was the state space reconstruction. Local dynamic stability of walking was quantified by estimating the average exponential rates of divergence of initially neighboring trajectories in state space as they evolve in real time. These local divergence exponents provide a direct measure of the sensitivity of the system to extremely small (i.e. local) perturbations. Positive exponents indicate local instability, with larger exponents indicating greater sensitivity to local perturbations. Nearest neighbor points on adjacent trajectories in the reconstructed state space represent the effects of small local perturbations to the system. Euclidean distances between neighboring trajectories in state space were computed as a function of time and averaged over all original pairs of initially nearest neighbors. Local divergence exponents were estimated from the slopes of linear fits to these exponential divergence curves:

1 $$y\left(i\right)=\frac{1}{\mathrm{\Delta t}}\langle \ln\;\mathrm{dj}\left(i\right)\rangle$$

where *dj(i)* is the Euclidean distance between the *j* th pair of initially nearest neighbors after *i* discrete time steps (i.e. *i* Δ*t* seconds) and 〈.〉 denotes the average over all values of *j*. Since the intrinsic time scales are different for each subject (i.e. different average stride times), the time axes of these curves were rescaled by multiplying by the average stride frequency for each subject. sLE were calculated from the slopes of linear fits to the divergence curve between 0 and 1 stride.

### Maximum Floquet Multipliers (FM)

The first step of orbital stability analysis via FM was the state space reconstruction. Two approaches were used: direct inclusion of acquired variables (acceleration time series) into the state space and delay-embedding reconstruction. Delay embedding is a technique to reconstruct a dynamical system from a sequence of observations. Standard embedding techniques were used [15]; an appropriate state space was reconstructed from each time series and its time delayed copies. An embedding dimension *d*_*E*_ = 5 was always chosen; many studies in literature agree in considering this to be an appropriate dimension for gait data [15, 24, 27, 37]. A fixed time delay *τ* = 10 was always used [27, 37].

Stride cycles were considered as the time between consecutive right heel strikes and were resampled to be 101 samples long, because Floquet theory assumes that the system is strictly periodic. A Poincaré section was defined at each percentage of the gait cycle (0% = right heel strike).

The Poincaré map:

2 $$S_{k+1}=F\left(S_{k}\right)$$

defines the evolution of the state *S*_*k*_ to the state *S*_*k+1*_ at each Poincaré section, for each stride k.

The limit cycle trajectory was defined as the average trajectory across all strides. This produces a fixed point in each Poincaré section:

3 $$S^{*}=F\left(S^{*}\right)$$

A linear approximation of Eq. (2):

4 $$\left[S^{k-1}-S^{*}\right]\approx J\left(S^{*}\right)\left[S^{k}-S^{*}\right]$$

allows calculating how system states diverge from or converge to fixed points. The FM are the eigenvalues of the Jacobian matrix J(S*). The maximum FM is believed to govern the dynamics of the system, and hence to be the most representative in terms of instability. FM was calculated for each Poincaré section (0 – 100% of the gait cycle). If the FM have magnitude < 1, the system remains stable, otherwise, the system tends to diverge from the limit cycle and become unstable. The overall mean value of FM across the gait cycle was calculated and used in the analyses.

### Recurrence quantification analysis (RQA)

The first implementation step of RQA was the reconstruction of the phase space by means of delay embedding. In this study, an embedding dimension of 5 and a delay of 10 samples were used, based on previous studies [24, 28, 37]. A distance matrix based on Euclidean distances between normalized embedded vectors was then constructed; the recurrence plot was obtained by selecting a radius of 40% of the max distance, and all cells with values below this threshold were identified as recurrent points. A radius of 40% was chosen [18].

A number of measures can then be obtained by RQA; in this study, RR, DET, averaged diagonal line length (AvgL) and maximum diagonal line length (MaxL) were calculated (Eq. 5, 6, 7, 8), reflecting different properties of the system.

5 $$\mathrm{RR}=\frac{1}{N^{2}}\sum_{i,j=1}^{N}R_{i,j}$$

where N is the number of points on the phase space trajectory;

6 $$\mathrm{DET}=\frac{\sum_{l=4}^{N}lP_{l}}{\mathrm{RR}}$$

where *l* is the length of diagonal lines, represented through a histogram (P_l_);

7 $$\mathrm{AvgL}=\frac{\sum_{l=4}^{N}lP_{l}}{\sum_{l=4}^{N}P_{l}}$$

8 $$\mathrm{MaxL}=\max\left(\left\{l_{i};i=1\ldots N_{l}\right\}\right)$$

where N_l_ is the number of diagonal lines in the recurrence plot.

### Multiscale entropy (MSE)

MSE was implemented constructing consecutively more coarse-grained time series; this procedure implies averaging increasing numbers of data points in non-overlapping windows of length *τ*. Sample entropy (SE) [21] was then calculated for each coarse-grained time series, in order to obtain entropy measures at different scales; SE quantifies the conditional probability that two sequences of *m* consecutive data points, similar to each other (distance of data points inferior to a fixed radius *r*) will remain similar when one more consecutive point is included, thus reflecting the regularity of the time series [20]. SE at each time scale *τ* is hence a function of *m* and *r*, and is expressed as the negative of the natural logarithm of the conditional probability *C(r)* that two sequences, that are close within a tolerance *rδ* for *m* consecutive points, remain close at the next point [22], where *δ* is the standard deviation of the original series:

9 $$\mathrm{SE}=‒\ln\frac{C^{m+1}\left(r\right)}{C^{m}\left(r\right)}$$

MSE was hence calculated for values of *τ* ranging from 1 to 6, *m* = 2 and *r* = 0.2, as suggested by Pincus [23] and later applied by Richman and Moorman to biological time series [21].

## Authors’ original submitted files for images

Below are the links to the authors’ original submitted files for images. Authors’ original file for figure 1 Authors’ original file for figure 2
