# Supplementary figures and images for: Measures of gait stability: performance on adults and toddlers at the beginning of independent walking
Source: J Neuroeng Rehabil. 2014 Sep 3;11:131. doi: 10.1186/1743-0003-11-131 (PMC4163161; doi:10.1186/1743-0003-11-131)

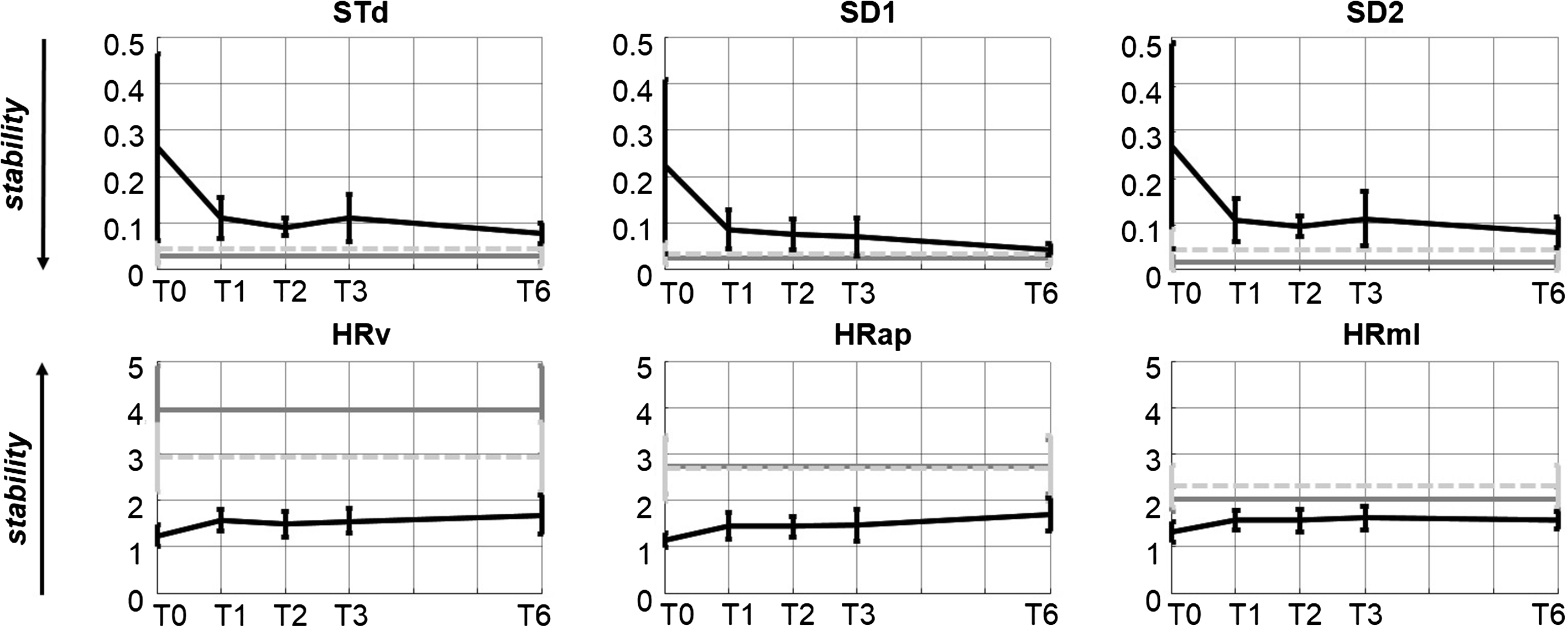

Supplement: Supplementary file 1 — Authors’ original file for figure 1 [file 12984_2014_653_MOESM1_ESM.tif]

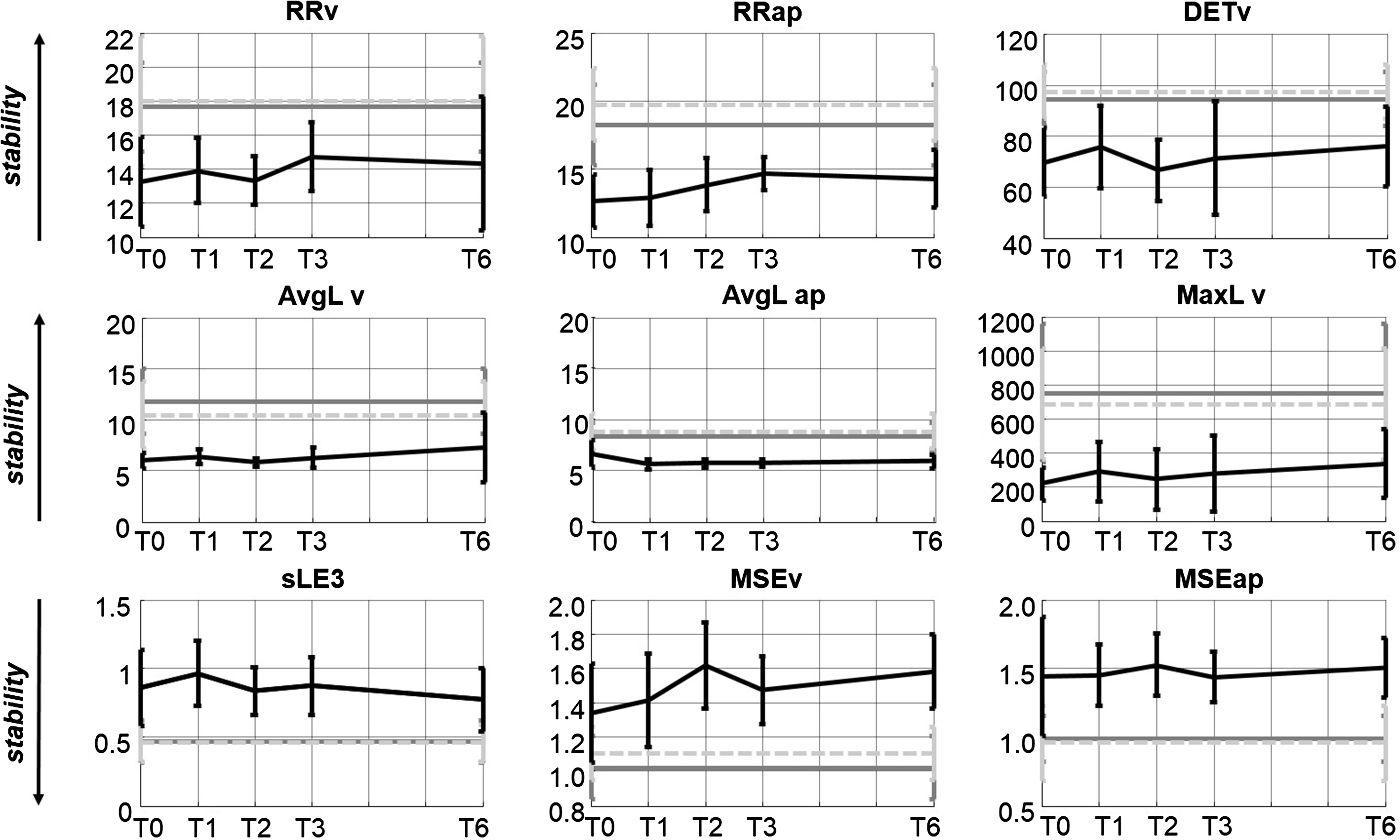

Supplement: Supplementary file 2 — Authors’ original file for figure 2 [file 12984_2014_653_MOESM2_ESM.tif]
